# Impact of Hexyl Branch Content on the Mechanical Properties and Deformation Mechanisms of Amorphous Ethylene/1-Octene Copolymers: A Molecular Dynamics Study

**DOI:** 10.3390/polym16233236

**Published:** 2024-11-21

**Authors:** Ruijun Zhang, Qiqi He, Hongbo Yu, Junhua Li, Yuexin Hu, Jianhua Qian

**Affiliations:** 1 College of Chemistry and Chemical Engineering, China University of Petroleum (East China), Qingdao 266555, China; zhangruijun523@gmail.com (R.Z.); qqhe65878@gmail.com (Q.H.); 2 PetroChina Fushun Petrochemical, Fushun 113001, China; yhbfssh@petrochina.com.cn; 3 College of Petrochemical Engineering, Liaoning Petrochemical University, Fushun 113001, China; lijunhua0521@163.com

**Keywords:** ethylene/1-octene copolymer, chain branching, molecular dynamics simulation, uniaxial tension, mechanical properties

## Abstract

Ethylene/1-octene copolymers exhibit enhanced flexibility and impact resistance compared to polyethylene, which makes them well suited for applications in advanced plastics and elastomers. United-atom molecular dynamics (MD) simulations were conducted to explore the mechanical behavior and deformation mechanisms of ethylene/1-octene copolymers under uniaxial tensile loading. This study systematically examined the influence of temperature, polymer chain length, chain quantity, and strain rate, with a specific focus on how hexyl branch content impacts the mechanical properties of amorphous ethylene/1-octene copolymers. The simulation results indicate that as the branch content increases, the yield strength and elastic modulus decrease, suggesting a trade-off between flexibility and mechanical strength. Energy decomposition analysis reveals that copolymers with more branched chains undergo greater changes in van der Waals energy. Additionally, as the branch content increases, the reduction in dihedral angle energy in the strain hardening region becomes more gradual, and the rate and the extent of the transition of dihedral angles from *gauche* to *trans* conformation decrease under deformation. Ethylene/1-octene copolymers exhibit higher chain entanglement parameters compared to linear polyethylene, with these parameters increasing as the branch content rises. Moreover, increasing the branch content results in a less pronounced increase in chain orientation along the loading direction.

## 1. Introduction

Ethylene-based polymers like polyethylene (PE) are widely used due to their versatility and strong mechanical properties [1,2]. However, the growing demand for materials with greater flexibility and impact resistance, while maintaining strength, has led to increased interest in ethylene/1-octene copolymers (POEs) [3,4]. POEs with more than 20% 1-octene, classified as polyolefin elastomers, have a higher amorphous content, enhancing their flexibility [1,5,6,7,8,9,10,11].

POEs with high short-chain branching (SCB) exhibit distinct properties compared to linear low-density polyethylene (LLDPE), sparking extensive research on the effects of branching on rheology [12,13], deformation, and mechanical performance [14,15]. He et al. [16] found that as the comonomer content increases, the strain softening during tensile testing gradually disappears, the yield stress decreases, and the elongation at break increases in the ethylene/1-octene copolymers. Mauler et al. [11] studied the effect of comonomer content on the dynamic mechanical properties of the ethylene/1-octene copolymer, finding that with higher comonomer content, the β-transition in dynamic mechanical behavior progressively increases. Stadler et al. [17] observed that differences in comonomer content notably influence the ethylene/1-octene copolymer’s rheological behavior; for instance, zero-shear viscosity (η₀) increases with rising comonomer content. Chum et al. [18] reported the tensile properties of ethylene/1-octene copolymers with varying comonomer content. The results showed that as comonomer content increased, the Young’s modulus and yield strength of the copolymer gradually decreased. Hiss [19] investigated the tensile stress–strain behavior of different polymers at room temperature and found that the strain strengthening modulus increased with the increase in crystallinity and entanglement degree. Kennedy [20,21] found that the degree of crystallinity, the degree of branching, and the molecular weight influence the strain strengthening stage in the tensile process. Mandelkern et al. [22,23,24] investigated how short-chain branching concentration (SCBC) and length (SCBL) affect the crystallization of branched semi-crystalline PE, finding that higher SCB content lowers crystallinity, melting temperature, and wafer thickness.

Molecular dynamics simulations have proven to be a reliable and promising tool for studying polymer materials at the atomic scale, offering insights that are often beyond the reach of experimental methods [25,26]. Due to the significant amorphous phases and the limited understanding of tensile deformation mechanisms in amorphous polymers, molecular dynamics (MD) studies on the microstructural evolution during their tensile deformation are of great importance. Previous studies on amorphous polyethylene under uniaxial tensile deformation identified four key stages: elastic deformation, yield deformation, strain softening, and strain strengthening. Brown [27] and Hossain et al. [28] examined these stages and the effects of factors such as temperature, chain number, and chain length on deformation. Similarly, Yashiro et al. [29] observed a conformation shift from *gauche* to *trans* during the yield stage. Shang et al. [30] and Bao et al. [31] further extended this research by studying the impact of structural parameters and cyclic loading on the mechanical response of amorphous polyethylene. Many MD studies have focused on the effect of branch concentration and length on the mechanical properties of amorphous polymers. Moyassari et al. [15] introduced short branches into PE using Monte Carlo simulations, finding that branching increased the interlayer connectivity and expanded the end-to-end distance of short chains. Liao et al. [32] showed that longer branches reduce equilibrium density but increase yield strength, while higher branch content has the opposite effect. Yan [33] explored the impact of branching and temperature on the mechanical and shape memory properties of branched PE. Moreover, MD simulations have also shown that SCB influences polymer crystallization. Zhang et al. [34,35,36,37] found that lower SCB content in single PE chains promotes crystallization and results in more regular lamellar structures, while Gao et al. [38,39] demonstrated that SCB concentration affects crystallization kinetics and morphology, with SCB length only influencing the final morphology.

**Scheme 1:**
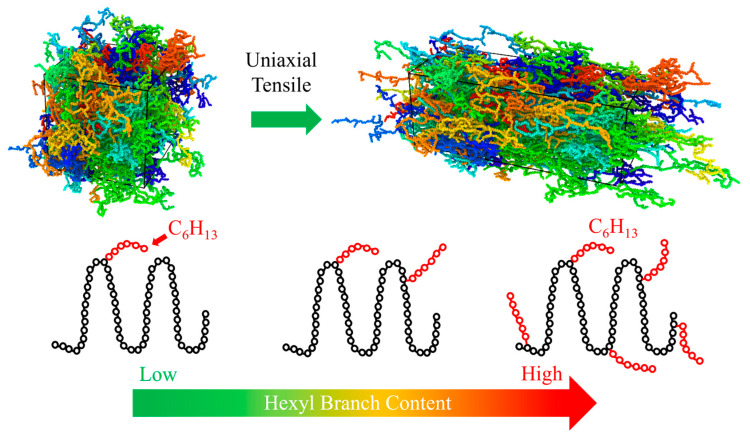
Schematic diagram showing the study of how varying hexyl branch contents affect the tensile behavior of POE systems through molecular dynamics uniaxial tension simulations.

While most MD studies focus on linear polyolefins, ethylene/1-octene copolymers are receiving more attention as high-performance materials. Investigating how hexyl branch content affects POEs during tensile deformation can provide valuable insights into their deformation mechanisms. In this work, amorphous ethylene/1-octene copolymer models with varying hexyl branch contents were designed. The mass content of the branches ranged from approximately 0 to 40%, and the hexyl branches were distributed at regular intervals along the main chain backbone. This study systematically examined how temperature, strain rate, polymer chain length, and quantity affect the copolymers, with particular emphasis on the influence of hexyl branch content on their mechanical behavior. Various energy components were evaluated to explore the deformation mechanisms under the influence of hexyl branches and changes in chain structure, such as bond length, bond angle, and dihedral angle. Additionally, the evolution of chain orientation and entanglement, which characterize structural changes during the tensile process, was investigated. Scheme 1 shows a schematic diagram showing the study of how varying hexyl branch contents affect the tensile behavior of POE systems through molecular dynamics uniaxial tension simulations.

## 2. Simulation Methodology

### 2.1. Force Field Selection

Amorphous ethylene/1-octene copolymers were simulated using a united atom (UA) model with the Dreiding potential [40], where -CH_2_- or -CH_3_ groups are treated as single particles. The UA model is advantageous for studying polymer systems with numerous monomers, significantly reducing computational time. Additionally, previous studies on polyethylene (PE) molecular simulations have extensively utilized the Dreiding potential, validating the model’s accuracy for ethylene-based polymers [28,30,31,41,42]. The Dreiding potential accounts for interactions in the united atom model through four components: bond stretching, angle bending, dihedral angle term, and van der Waals forces. The total potential energy of the system can be expressed as follows:

(1) $$E_{\mathrm{total}}=E_{\mathrm{bond}}+E_{\mathrm{angle}}+E_{\mathrm{dihedral}}+E_{\mathrm{non}-\mathrm{bonded}}$$

where *E*_bond_, *E*_angle_, and *E*_dihedral_ make up bonded terms, and *E*_non-bonded_ is the van der Waals potential. The bonded potentials are represented by the following expression:

(2) $$\begin{array}{l} E_{\mathrm{bond}}=\frac{1}{2}K_{\mathrm{bond}}{\left(r-r_{0}\right)}^{2}\\ E_{\mathrm{angle}}=\frac{1}{2}K_{\mathrm{angle}}{\left(\theta -\theta_{0}\right)}^{2}\\ E_{\mathrm{dihedral}}=\sum_{i=0}^{3}K_{i}{\left(\cos\varphi \right)}^{i} \end{array}$$

where *K*_bond_ and *K*_angle_ represent the stiffness parameters for bond stretching potential and bond angle bending, while *r*_0_ and *θ*_0_ denote the equilibrium values of bond length and bond angle. The coefficients *K_i_* (*i* = 0–3) correspond to the dihedral potential term. The van der Waals energy is expressed as follows:

(3) $$E_{\mathrm{non-bonded}}=4\varepsilon \left[{\left(\frac{\sigma }{r}\right)}^{12}-{\left(\frac{\sigma }{r}\right)}^{6}\right],\;r\leq r_{c}$$

In the Lennard–Jones potential, the parameter σ represents the distance at which the interatomic force is zero, while ε corresponds to the depth of the potential energy well between two atoms. *r* denotes the separation between particles, with *r*_c_ being the cutoff distance set to 10 Å in the present simulations. Table 1 provides a detailed listing of the force field and its corresponding parameters [28,31,43]. The structural parameters of polyethylene, such as bond length and bond angle, obtained using these simulation parameters closely match the experimental values [44], confirming the reliability of the parameters used in this work.

### 2.2. Model Construction

Ethylene/1-octene copolymer (POE) is produced by polymerizing ethylene with 1-octene. The polymer has -CH_2_- groups in the main chain and typical hexyl groups (-[-CH_2_-CH_2_-CH_2_-CH_2_-CH_2_-CH_3_]) as branched chains. These hexyl groups are distributed on the copolymer backbone (-[-CH_2_-CH_2_-]_m+n_-), with *m* and *n* indicating the number of ethylene monomers and 1-octene comonomers, respectively. In this study, the amorphous POE models with three different levels of hexyl branching were developed. Linear polyethylene (PE) chains, composed solely of ethylene monomers, were also modeled as a reference without branching. In addition, the amorphous POE models varying in chain length and number, but with consistent branched chain content, were generated to investigate their effects. Various categories were arranged to explore the influence of chain lengths, number of chains, and hexyl branched chain content, as shown in Table 2. Note that *n*_BC_ represents the hexyl branch count per POE chain. *l*_c_ signifies the total count of united atoms in a POE backbone, while *c*_BC_ represents the ratio of united atoms in all branched chains relative to the entire backbone. This ratio characterizes the content of hexyl branched chains. POE systems are classified according to chain quantity, backbone atom count, and branched chain content. For instance, 60POE650/23 means that the system has 60 chains with 650 united atoms in the backbone of each chain and a branched chain content of 23%.

The initial POE system was prepared via the Monte Carlo method with a self-avoiding random walk approach [45]. Initially, each chain’s starting atom was positioned at a lattice site, and the chain then extended in a designated direction. This growth occurred incrementally and probabilistically, taking into account bond length and available sites within the lattice. To generate a hexyl branched chain, it advanced six steps forward when the chain reached the first branching point, then retraced those six steps back to the branching point. Afterward, the molecular chain changed direction and continued to grow, thereby forming the desired side chain. It is worth noting that the side chains are distributed along the backbone at fixed intervals. Periodic boundary conditions were utilized in every direction of the simulation box. For statistical analysis, three different configurations were established for each system listed in Table 2 to eliminate the influence of specific initial structural randomness.

In this study, the POE model is established as amorphous rather than semi-crystalline. This choice is primarily because many ethylene/1-octene copolymers exhibit amorphous or low crystallinity characteristics in practical applications, especially when the comonomer content is high, which can inhibit crystallization. The amorphous model better reflects the mechanical response of the real amorphous regions and more effectively captures the impact of branch content on the mechanical properties of the copolymer. Therefore, the current modeling approach is considered appropriate for the study’s focus. The effects of the crystalline phase, however, are recommended to be explored in a separate investigation.

### 2.3. Simulation Details

**Figure 1:**
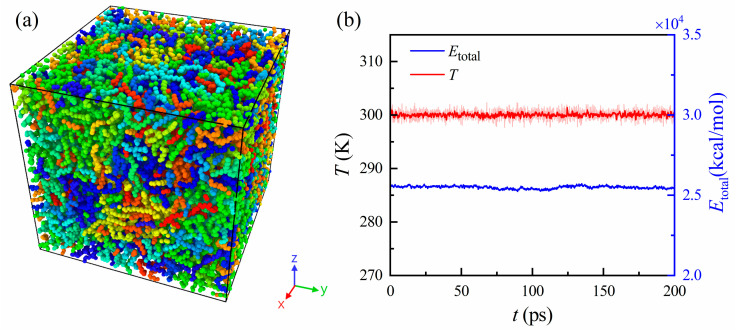
(**a**) A representative system 60POE650/42 model after equilibrium in 300 K; (**b**) the variations in temperature and total energy of 60POE650/42 during the end of the relaxation process.

The MD simulations were carried out using Large-scale Atomic/Molecular Massively Parallel Simulator (LAMMPS, Version 2 Aug 2023, Sandia National Laboratories, Albuquerque, NM, USA) [46], and all molecular visualizations were performed with Open Visualization Tool(OVITO, Version 3.10.6, 2024, Materials Science Software, Stuttgart, Germany), an open-source visualization and analysis tool for particle-based simulations [47]. The initial system was subjected to energy minimization to eliminate any unreasonable contacts. Then the system was first equilibrated using the NVT ensemble at 600 K for 100 ps. Subsequently, it underwent further relaxation under the NPT ensemble at 600 K and 1 bar over 5 × 10^5^ timesteps with a timestep of 1 fs, with pressure controlled by the Berendsen barostat. Afterward, an annealing process was performed to relieve residual stress, ranging from 600 K to the desired temperature, with two cycles over 5 × 10^5^ timesteps at each stage. The simulation involved was then cooled from 600 K to the target temperature over approximately 500 ps in the NPT ensemble, followed by further relaxation at the desired temperature of 500 ps. A 1 fs timestep was used in all MD simulations, and the Nose–Hoover method regulated the system temperature. As an example, the POE system 60POE650/42 listed in Table 2 is shown at equilibrium at 300 K in Figure 1. The cell size was about 113.08 × 113.08 × 113.08 Å^3^. Additionally, the system temperature stabilized at 300 K and the total energy *E*_total_ was well maintained at approximately 2.5 × 10^4^ kcal/mol during the last 200 ps of relaxation, which proved that the relaxation process mentioned above is reliable for reaching the equilibrium.

After reaching equilibrium, tensile deformation was introduced by applying uniaxial tension at a steady deformation rate in three directions, while maintaining a 1 bar pressure in the orthogonal directions. Given the amorphous nature of the POE systems, tensile behavior along these three directions is expected to be similar. To minimize simulation errors, data from these directions were averaged during subsequent data processing. The NPT ensemble was employed throughout the uniaxial tensile process.

## 3. Results and Discussion

**Figure 2:**
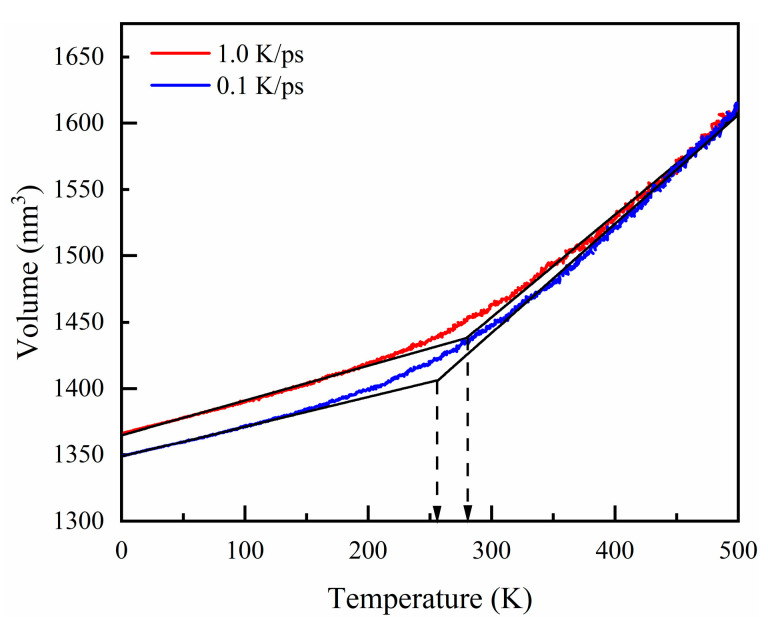
Volume change with temperature ranging from 1 to 500 K for the 60POE650/42 model.

Temperature has a significant impact on POE’s mechanical behavior. The glass transition temperature *T*_g_, an inherent property of amorphous polymer materials reflecting the shift from a rigid to a more rubber-like state, was determined based on the temperature dependence of volume [28,30,31,48,49]. The POE system was initially equilibrated at 500 K and before being gradually cooled to 1 K at rates of 1.0 K/ps and 0.1 K/ps, respectively. Figure 2 illustrates how the volume of the 60POE650/42 system varies with temperature. The *T*_g_ of the POE system was estimated by identifying the intersection point of the two linear fits over the 1–200 K and 400–500 K ranges. In Figure 2, the estimated glass transition temperatures for the 60POE650/42 system are approximately 255 K and 282 K, respectively. These values are consistent with earlier MD simulations and experiment findings [28,30,31,50,51]. The cooling rate was also observed to affect the predicted *T*_g_. In addition, the *T*_g_ of other systems with varying chain lengths, numbers of chains, and hexyl branch contents do not differ significantly from those of the 60POE650/42 system.

According to Figure 2, 100 K corresponds to the glassy state, while 250 K and 300 K fall within the transition zone between glassy and rubbery states. The temperature of 400 K corresponds to the rubbery state. Thus, 100 K, 250 K, 300 K, and 400K were selected for further investigation to examine the temperature dependence of the mechanical properties of POE. By studying these temperatures, we can better understand how POE behaves under different thermal conditions, providing insights into its performance across various states.

### 3.1. Stress–Strain Behavior Under Different Conditions

#### 3.1.1. Influence of Chain Length, Chain Count, and Hexyl Branch Content

**Figure 3:**
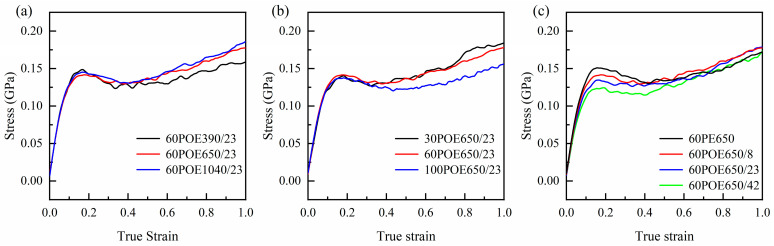
Stress–strain relationships of POE with (**a**) varying chain lengths, (**b**) different numbers of chains, and (**c**) different branch contents at 300 K and 10^10^ s^−1^, respectively.

The POE systems were simulated under uniaxial tensile loading at 300 K to examine the effect of chain length on tensile properties. The models had chain lengths of *l*_c_ = 390 (*M*_w_ = 6730 g·mol^−1^), 650 (*M*_w_ = 11,216 g·mol^−1^), and 1040 (*M*_w_ = 17,946 g·mol^−1^). Figure 3a depicts four distinct stages in the stress–strain curves: elastic, yield, strain softening, and strain hardening, commonly observed in polymers [28,52]. In the elastic region, stress rises nearly in proportion to the applied strain. Once the yield point is reached, the stress drops to a trough, reflecting polymer relaxation. Subsequently, stress begins to rise again, leading to strain hardening. In Figure 3a, it can be observed that longer chain lengths exhibit higher strain hardening stress. The increase in polymer chain length leads to a higher entanglement density and more extensive interchain entanglements, resulting in reduced chain mobility. As a result, the motion of the chain segments is more constrained under external stress. On the stress–strain curve, the polymer systems with longer chains show more pronounced strain hardening behavior, meaning that at higher strains, these systems demonstrate greater resistance to deformation.

Three POE systems listed in Table 2, with 30, 60, and 100 chains, were selected to explore how varying chain numbers impact the mechanical characteristics of POE. The stress–strain behavior for POE systems remains relatively consistent across different chain counts at 300 K in Figure 3b. Notably, the stress–strain relationship smooths out with an increasing chain count, as demonstrated by Hossain et al. [28]. This smoother behavior indicates that the system more closely approximates real-world behavior. Therefore, systems with 60 molecular chains were used to examine the mechanical properties of POE in the subsequent simulations.

Four POE systems with varying branch contents were tested to determine how branch content influences the tensile properties. Figure 3c presents the stress–strain curves of POE models with varying branch contents at 300 K. Young’s modulus and ultimate yield stress are reduced as the hexyl branch content in the POE system increases. An inverse relationship between strength and branch content is observed in the POE systems. Branched chains add complexity to the molecular structure, making the polymer more flexible during stretching and reducing its stiffness. Additionally, branched chains weaken the interactions between polymer chain segments, resulting in a lower maximum stress that the material can withstand before reaching its yield point.

**Figure 4:**
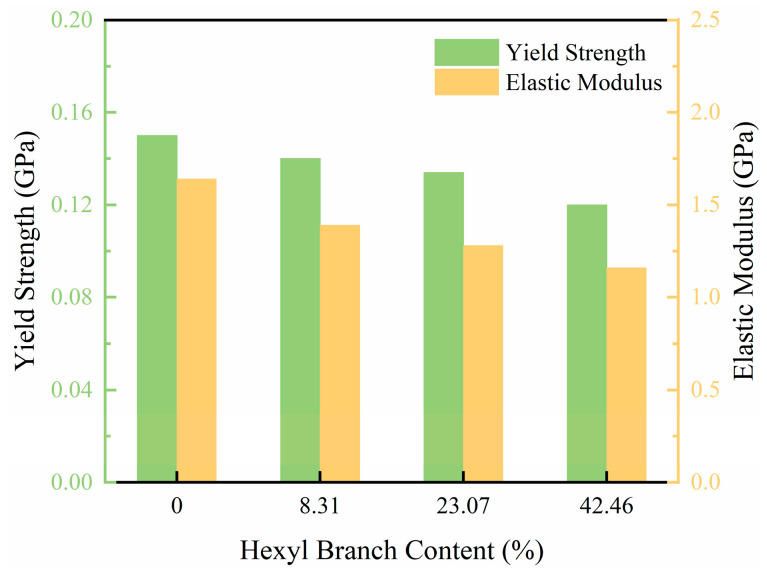
Comparison of the yield strength and elastic modulus of POE with different hexyl branch contents at 300 K and 10^10^ s^−1^.

Based on the stress–strain curves, the elastic modulus and yield strength of POE are calculated to quantitatively analyze the influence of various factors on its tensile properties. Figure 4 shows the comparison of the yield strength and elastic modulus of POE systems with different hexyl branch contents at 300 K and 10^10^ s^−1^. The results show that increasing the hexyl branch content decreases both the elastic modulus and yield strength of the POE system. Specifically, as the hexyl branch concentration increases from 0 wt% to 40 wt%, the elastic modulus decreases from 1.64 GPa to 1.16 GPa (a reduction of 29.27%), while the yield strength decreases from 0.15 GPa to 0.12 GPa (a reduction of 20%). It can be seen that the reduction in yield strength is relatively minor, which aligns with studies showing that branching primarily reduces elasticity without significantly affecting the material’s resistance to yielding. Additionally, Figure 4 shows that the elastic modulus and the yield strength of 60PE650 (linear PE) are 1.64 GPa and 0.15 GPa, respectively. These values align with previous MD simulations [28,31,49] but are higher than the experimental data [16], likely due to the higher strain rates in MD simulations. Compared to other branched POE systems, its regular structure promotes stronger intermolecular interactions, resulting in higher rigidity and strength.

To indicate the differences from the real system, a comparison is made with experimental data from Reference [16]. Table 3 presents the elastic modulus and yield strength of POE systems with different branch concentrations under the current simulation and real experimental conditions. In the current simulation, as the hexyl branch concentration increases from 8.31 wt% to 42.46 wt%, the reduction rate of the elastic modulus is 6.73 MPa/wt%, meaning that for each 1% increase in branch concentration, the elastic modulus decreases by 6.73 MPa. The reduction rate of the yield strength is 0.62 MPa/wt%. In real experiments, as the branch concentration increases from 15.02 wt% to 24.57 wt%, the reduction rates for the elastic modulus and the yield strength are 3.11 MPa/wt% and 0.34 MPa/wt%, respectively—about half of the values in the current simulation. Although the reduction rates for the elastic modulus and yield strength in the simulation are slightly higher than the experimental data, both show similar trends, indicating that the simulation can capture the effect of branch concentration on mechanical properties to some extent and has predictive value.

#### 3.1.2. The Role of Temperature and Strain Rate in Mechanical Response

**Figure 5:**
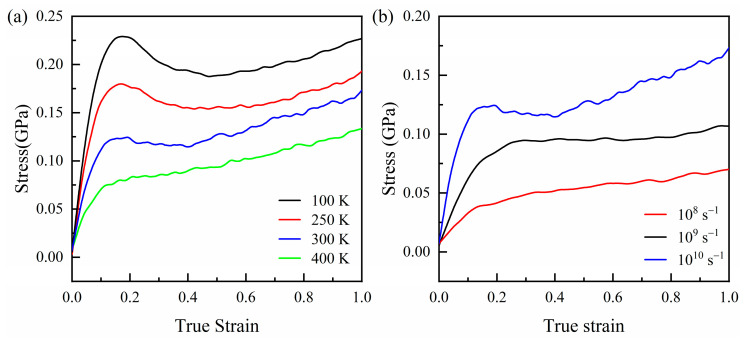
(**a**) Temperature-related changes in the stress–strain profile of the 60POE650/42 system under a deformation rate of 10^10^ s^−1^, evaluated at 100 K, 250 K, 300 K, and 400 K. (**b**) Stress versus strain curves of the 60POE650/42 system across various strain rates at 300 K.

The stress–strain behavior of the 60POE650/42 (*M*_w_ = 12,898 g·mol^−1^) system at different temperatures was investigated at 10^10^ s^−1^. It was noted in Figure 5a that the strength of POE weakens with rising temperatures, which aligns with expectations. Additionally, the classic four regimes of a polymer material’s stress–strain curve are still evident at 100 K, 250 K, and 300 K. However, at 400 K, the curve becomes smoother, the point of yielding is less distinct, and no strain softening region is visible.

Uniaxial tension simulations were conducted on the 60POE650/42 model at 300 K with three strain rates of 10^8^, 10^9^, and 10^10^ s^−1^ to investigate how strain rate affects the POE system. Figure 5b and Table 4 illustrate that the elastic modulus of the 60POE650/42 model increases with higher strain rates. Similarly, the peak yield stress also rises with increasing strain rates, indicating a strain rate dependency. At a low strain rate of 10⁸ s⁻¹, the mechanical response curve does not fully show the four typical regions of polymer materials, as the curve is less pronounced. The elastic modulus and the yield strength calculated from simulations at different strain rates and temperatures are given in Table 4.

### 3.2. Energy Evolution During Stretching

#### 3.2.1. Temperature and Strain Rate Dependence

Section 2.1 outlines the total potential energy of the POE system comprising four energy terms: *E*_bond_, *E*_angle_, *E*_dihedral_, and *E*_non-bonded_. Each energy term accurately reflects changes in molecular conformation and can thus be used to account for the evolution of various interactions within the POE system.

**Figure 6:**
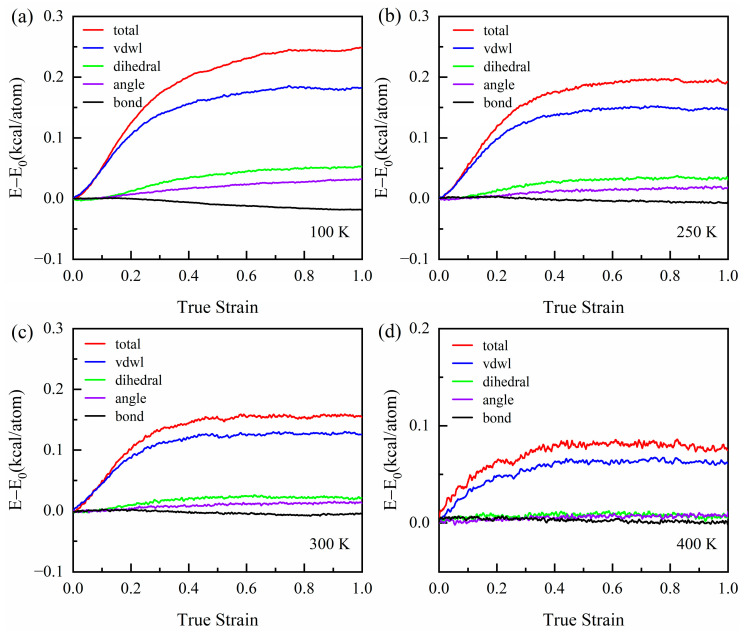
The individual energy terms variation of the 60POE650/42 system during tensile deformation at 10^10^ s^−1^ for temperatures (**a**) 100 K, (**b**) 250 K, (**c**) 300 K, and (**d**) 400 K, respectively.

Figure 6a–d illustrate how overall and component energy terms evolve for the 60POE650/42 system during the tensile simulation at 10^10^ s^−1^ and four different temperatures. To show the changes in individual energy terms during the uniaxial tension process, *E*−*E*_0_ was calculated for the total energy and for each of four energy terms, where *E* represents the energy during stretching and *E*_0_ is the initial energy, as depicted in Figure 6a–d. It can be found that the trends of individual energy terms at different temperatures are generally similar. In the elastic stage, the changes in *E*_bond_, *E*_angle_, and *E*_dihedral_ remain nearly zero, while the total energy curve closely follows the non-bond energy curve, which increases significantly. This indicates that the elastic region’s van der Waals effects between polymer chains are essential. After the elastic deformation stage, changes occur in bonding and dihedral angle components. The adjustment in *E*_bond_ is due to bond lengths moving toward their equilibrium state. *E*_dihedral_ initially rises in the strain softening region, followed by a gradual decline during the strain strengthening phase, and eventually stabilizes. This process involves the POE’s dihedral angle from a *gauche* arrangement to a low-energy *trans* conformation. The transformation from *gauche* to *trans* conformation under tensile loadings is also discussed by Chen et al. [53]. In Figure 6a–d, lower temperatures cause much greater changes in *E*_total_, *E*_non-bonded_, *E*_bond_, and *E*_dihedral_. In addition, except at 100 K, *E*_angle_ shows less variation than the other energy terms at 250 K, 300 K, and 400 K.

**Figure 7:**
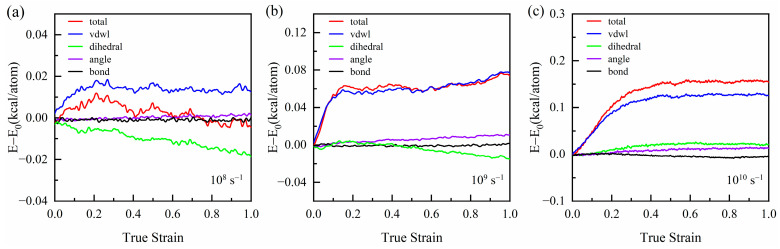
The individual energy terms variation of the 60POE650/42 system during tensile deformation at 300 K for strain rates of (**a**) 10^8^ s^−1^, (**b**) 10^9^ s^−1^, and (**c**) 10^10^ s^−1^.

Simulations were carried out on the 60POE650/42 model at 300 K to explore the influence of varying strain rates on energy evolution. The findings reveal that the strain rate strongly affects energy changes in the system. Figure 7a illustrates that *E*_dihedral_ decreases steadily with strain at lower strain rates, resulting in a less dramatic increase in total energy compared to that at higher strain rates. In Figure 7b, *E*_dihedral_ shows a slight increase during the elastic regime before dropping rapidly, causing the total and non-bond energy curves to overlap. In Figure 7c, both *E*_total_ and *E*_non-bonded_ show substantial changes with increasing strain at 10^10^ s^−1^, with *E*_total_ consistently surpassing *E*_non-bonded_. This is reflected in the graph where the total energy curve is positioned above the non-bond energy curve.

#### 3.2.2. Content of Hexyl Branched Chains Dependence

**Figure 8:**
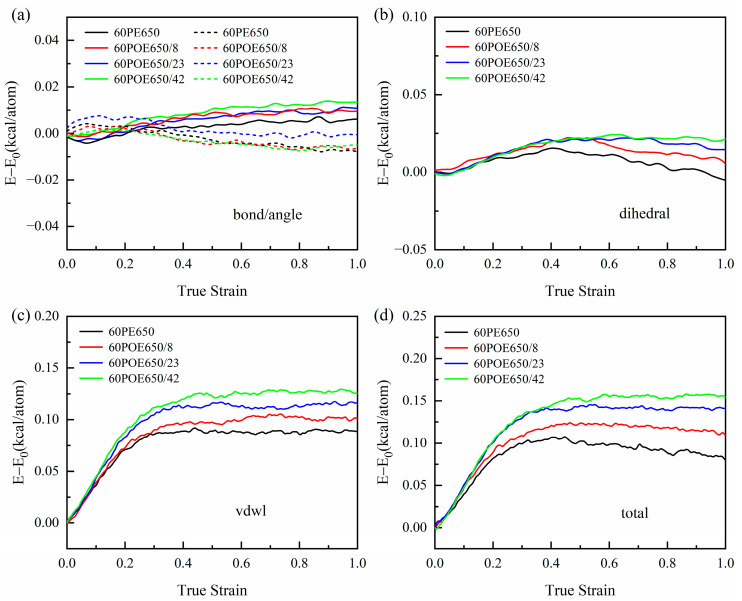
Variation in individual energy terms in POE systems with different hexyl branched chain contents during tensile deformation at 300 K and 10^10^ s^−1^. The evolution of (**a**) *E*_bond_ (----) and *E*_angle_ (—), (**b**) *E*_dihedral_, (**c**) *E*_vdwl_, and (**d**) *E*_total_ for POE systems with different branched contents.

Figure 8 shows the evolution of energy terms in POE systems with different branched chain contents during tensile simulations at 300 K and 10^10^ s^−1^. The variations in the energy terms for POE systems with varying branched chain contents follow generally consistent trends. As depicted in Figure 8a, *E*_bond_ and *E*_angle_ exhibit minimal change compared to the other energy terms, regardless of the branched chain content. In addition, the bond energy increases initially, followed by a gradual decrease as the bond lengths approach their equilibrium values. In contrast, the bond angle energy decreases initially, then gradually increases and stabilizes. Figure 8b presents that *E*_dihedral_ rises in the elastic and strain softening regions, before declining slowly during the strain hardening region. Although the dihedral angle energy changes similarly across systems with different branched chain contents through the initial deformation stages, a higher branched chain content leads to a smaller decrease in *E*_dihedral_ in the strain hardening regime. The main reason is that POE systems with a higher proportion of branched chains have more dihedral angles. When the branched chain content is higher, the flexibility of the molecular chains increases, allowing the system to absorb stress through more dihedral angle adjustments during stretching. Consequently, even though the deformation in the strain hardening stage is more pronounced, the more complex molecular chain structure can disperse and offset some of the energy through additional dihedral angle changes, resulting in a smaller decrease in dihedral angle energy.

In Figure 8c, POE systems with a higher number of branched chains experience greater alterations in *E*_vdwl_ during stretching due to the increased intermolecular interactions and complexity introduced by the branched chains. As the molecular chains are stretched and rearranged under external force, systems with more branched chains undergo more pronounced molecular displacements and structural changes. This structural alteration causes larger variations in van der Waals interactions at different stages. Specifically, the increase in branched chains shortens intermolecular distances, enhancing van der Waals interactions, which may result in an initial energy increase. Additionally, in the strain hardening stage, molecular chains with abundant branched chains require more space for rearrangement, leading to significant changes in van der Waals energy. As shown in Figure 8d, systems with a higher content of branched chains exhibit more significant changes in total potential energy, with the complex structure causing the potential energy to decrease more gradually during the strain hardening stage.

### 3.3. Development of Internal Configuration

#### 3.3.1. Variation in Bond Length, Bond Angle, and Dihedral Angle

**Figure 9:**
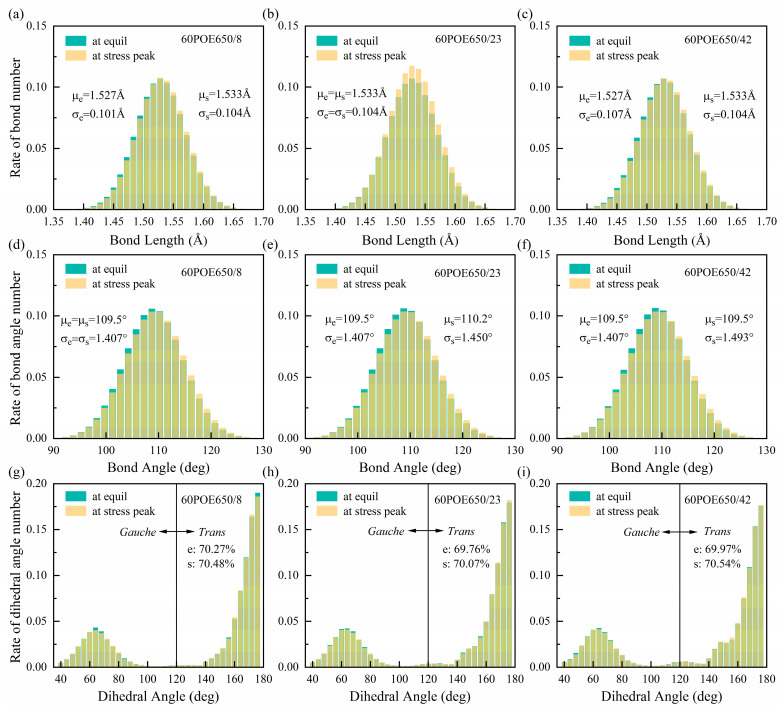
Profiles of bond length, bond angle, and dihedral angle for POE systems with different contents of hexyl branched chains at equilibrium (green bar) and at stress peak (light yellow bar) at 300 K and 10^10^ s^−1^.

Figure 9 illustrates the structural characterization of POEs with varying hexyl branch content, analyzed by bond length (top row), bond angle (middle row), and dihedral angle (bottom row) at equilibrium and at the stress peak (true strain = 0.2). The vertical axis represents the ratio of each component to the total. μ represents the mean, and σ denotes the standard deviation for each component. To calculate the proportion of *trans* conformers in the dihedral angles, a threshold of 120° was chosen, with dihedral angles greater than 120° classified as *trans* conformations.

The average bond length for all POE systems at equilibrium and at the stress peak was approximately 1.53 Å in Figure 9a–c, while the average bond angle was about 109.5°, as depicted in Figure 9d–f. These values closely match the equilibrium potential parameters *r*_0_ and *θ*_0_ described in Section 2.1. The simulated bond length and bond angle values are also in excellent agreement with the C−C bond distance and C−C−C intrachain bond angle of polyethylene measured by Caminiti et al. [44] using X-ray powder diffraction. When comparing the μ and σ values between the equilibrium state and the first stress peak in Figure 9, the bond length and bond angle distributions exhibit minimal change. In addition, there is no significant difference in the average bond length and angular configurations among POE systems with varying branched chain contents. These results support previous findings that bond lengths and angular parameters play a minor role during elastic deformation, with non-bonded interactions between molecular chains being more influential. This conclusion is consistent with the energy contributions of bond lengths and angular factors, as discussed in Section 3.2, which also show little change with increasing strain.

**Figure 10:**
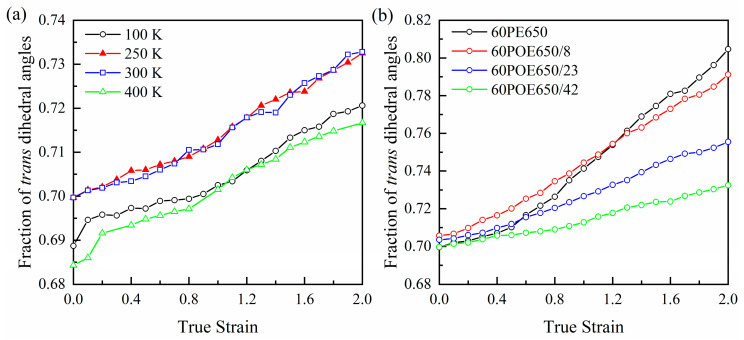
(**a**) The evolution of dihedral angles with strain in the 60POE650/42 system at multiple temperatures and 10^10^ s^−1^. (**b**) The evolution of dihedral angles with strain in POE systems with varying branched chain contents at 250 K and 10^10^ s^−1^.

Figure 9g–i display the distribution of dihedral angles for POE systems with different contents of branched chains at equilibrium and at the stress peak. The *gauche* conformation is mainly concentrated around 66°, while the *trans* conformation is more concentrated around 180°. Before stretching, the dihedral angles predominantly consist of *gauche* and *trans* conformations. The *gauche* conformation gradually decreases, with more dihedral angles shifting towards the trans conformation at the first stress peak. Moreover, Section 3.2 highlights that the variation in dihedral angle energy is essential to the deformation process of POE. Therefore, Figure 10 presents additional investigations into the variation in dihedral angles with strain at different temperatures and their evolution in POE systems with varying branched chain contents. Figure 10a shows that the limited flexibility of the POE chains leads to minimal changes in dihedral angle conformation at 100 K. As the temperature approaches *T*_g_, the increased chain mobility leads to a more significant shift from the *gauche* to the *trans* conformation. However, at 400 K, the increase in *trans* conformations is less pronounced than near *T*_g_. This may be due to the heightened thermal disturbances at higher temperatures, which introduce greater transient disorder and cause more frequent transitions between the *gauche* and *trans* conformations. As shown in Figure 10b, with increasing branched chain content in POE systems, both the rate and the extent of the transition of dihedral angles to the *trans* conformation decrease with increasing deformation. This is primarily due to the increased complexity of the polymer chains and the restricted space for free rotation imposed by the branched chains. The dihedral angle change curve of linear polyethylene (60PE650) has the steepest slope, indicating that the molecular chains in the linear polymer are more flexible, allowing the dihedral angles to adjust more rapidly to the *trans* conformation.

#### 3.3.2. Evolution of Free Volume

**Figure 11:**
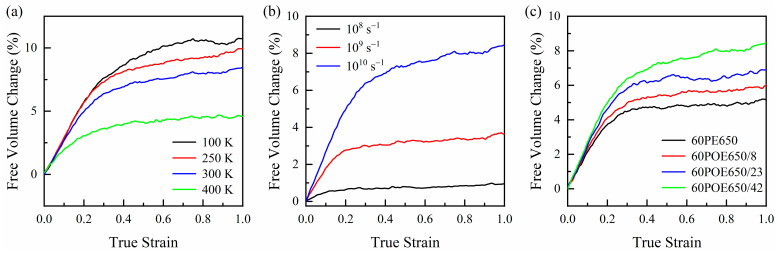
(**a**) Free volume changes in response to strain in the 60POE650/42 system at four different temperatures and 10^10^ s^−1^; (**b**) the evolution of free volume with strain in the 60POE650/42 model at 300 K and three different strain rates. (**c**) Free volume changes in response to strain in POE systems with varying branched chain contents at 300 K and 10^10^ s^−1^.

In MD simulations, changes in free volume during the deformation process of a polymer can reflect the material’s structure, mechanical properties, and deformation mechanisms. Changes in the simulation box volume can serve as an approximate indicator of variations in free volume. Figure 11a presents how free volume changes with strain for the 60POE650/42 model under four temperature conditions. It can be observed that across different temperatures, free volume initially rises sharply in response to strain and then remains almost unchanged. However, the higher the temperature, the smaller the increment in free volume. Figure 11b shows how different strain rates affect free volume changes at rates of 10^8^ s^−1^, 10^9^ s^−1^, and 10^10^ s^−1^. At 10^8^ s^−1^, the free volume shows no significant change. During the early stages, the free volume grows in response to strain, with higher strain rates leading to more significant changes. Figure 11c illustrates the variation in free volume relative to strain in POE systems with varying branched chain contents at 300 K and 10^10^ s^−1^. Free volume increases rapidly when strain increases until it reaches approximately 30%, after which the change becomes negligible. In addition, with increasing branch content, the change in free volume during the stretching process shows a more pronounced increase.

#### 3.3.3. Transformation in Chain Orientation

In polymer materials, chain orientation refers to the alignment of molecular chains in a specific direction under the influence of external forces, which significantly impacts the material’s final properties. Chain orientation is a key indicator for characterizing the microstructure of polymer segments during deformation. The method described in the literature [28,54] is used here to introduce a chain orientation parameter that represents the alignment of molecular chains with the calculation formula given in Equation (4).

(4) $$P_{2}=\frac{3}{2}\langle \cos^{2}\theta \rangle -\frac{1}{2}$$

*θ* is defined as the angle every bond creates relative to the tensile axis, 〈·〉 denotes the mean value across all segments of the chains, and the final statistical value of *P*_2_ represents the average orientation factor of all chemical bonds in the system at a certain time. If *P*_2_ = 1, it indicates that all chemical bonds are aligned with the tensile direction. If *P*_2_ = 0, it means that the chemical bonds are randomly oriented. Conversely, *P*_2_ = −0.5 signifies that every chemical bond is aligned orthogonally to the tensile axis.

**Figure 12:**
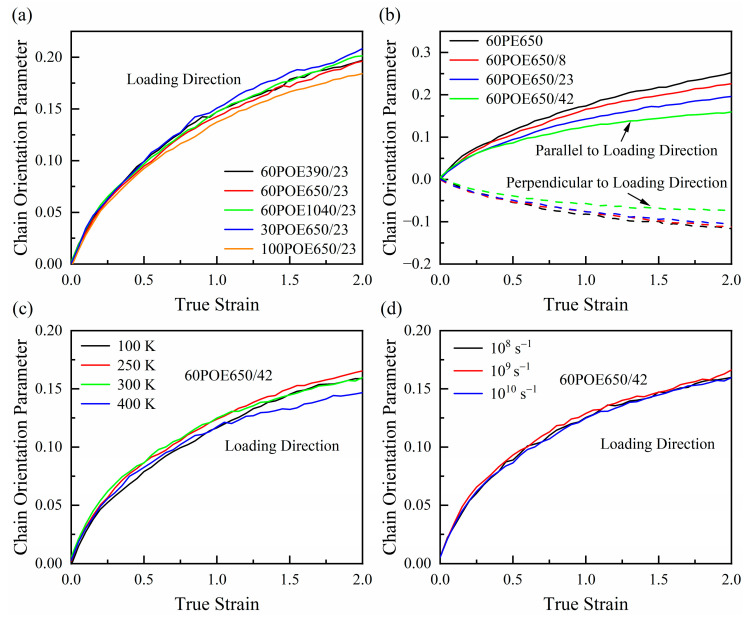
(**a**) Chain orientation changes with strain for various chain lengths and counts at 300 K and 10^10^ s^−1^; (**b**) the influence of branch contents on the chain orientation parameter at 300 K and 10^10^ s^−1^; (**c**) changes in chain orientation in response to strain in the 60POE650/42 system at four varied temperatures and 10^10^ s^−1^; (**d**) chain orientation changes in response to strain with varying strain rates.

As shown in Figure 12, as expected, chain segments increasingly align with the direction of applied load as strain intensifies. Figure 12a further illustrates that chain quantity influences the orientation parameter. Specifically, after the strain softening stage, having more chains in the system leads to a reduced degree of orientation. Additionally, chain length does not significantly impact the orientation parameter. In Figure 12b, higher branched content reduces chain orientation. The presence of branched content increases the bending and twisting of the main chain, hindering its ability to fully orient along the tensile axis under stress. In the strain softening stage, the chain orientation of linear polyethylene (60PE650) increases rapidly and remains high during the strain hardening stage. For branched POE, the chain orientation increases more slowly in the strain softening stage and continues to increase gradually in the hardening stage, exhibiting greater orientation limitations. Figure 12c illustrates how temperature affects the chain orientation parameter at 10^10^ s^−1^. When the temperature is 300 K or lower, there is no significant difference in the chain orientation of the 60POE650/42 system. However, at 400 K, the chain orientation parameter decreases after the strain hardening stage. Last, the strain rate does not directly affect the magnitude of the chain alignment parameter, with minimal differences observed in the chain orientation parameters across different strain rates in Figure 12d.

#### 3.3.4. Variation in the Entanglement Parameter

Chains intertwine and disentangle under external loads, significantly influencing their mechanical performance. Here, we reference the method developed by Yashiro et al. [29] for calculating the entanglement angle to evaluate the entanglement parameter. The entanglement angle is defined as the angle made by the vector connecting the *i*-th atom to the (*i*−10)-th atom in the same molecular chain and the vector connecting the *i*-th atom to the (*i*+10)-th atom. This geometric characterization method is well consistent with the entanglement molecular weight of polyethylene. The entanglement angle of atoms is defined by Equation (5).

(5) $$\theta_{i}=\frac{e_{i+10}\cdot e_{i-10}}{\left|e_{i+10}\right|\left|e_{i-10}\right|}$$

An atom *i* is classified as entangled if its entanglement angle is below 90°. Consequently, the entanglement parameter of the POE system is determined by dividing the number of entangled atoms by the total number of atoms in the system.

**Figure 13:**
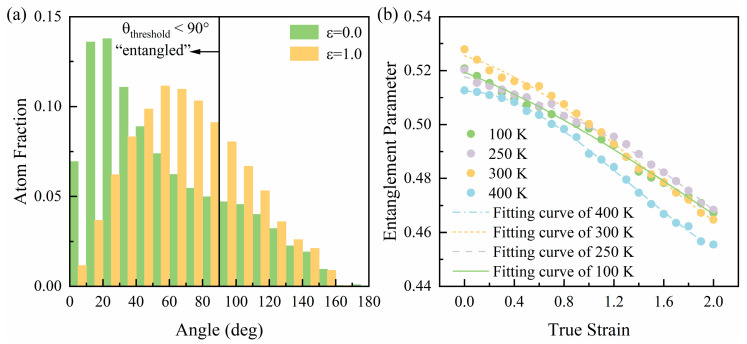
(**a**) The entanglement angle profile for the 60POE650/42 system at strain ε = 0 and ε = 1, respectively, under tensile stress at 300 K and 10^10^ s^−1^; (**b**) chain entanglement changes with strain for the 60POE650/42 system at various temperatures.

Figure 13a displays the angle distribution for 60POE650/42, as determined by the previously described method, at both equilibrium and ε = 1. The number of entangled atoms before deformation is expected to be higher than that after deformation in Figure 13a. In the initial state, the chains are relatively relaxed, and the number of entangled atoms is relatively stable. As the tensile strain increases, the polymer chains stretch, causing a decrease in the number of intrachain entanglements. Figure 13b illustrates how chain entanglement varies with strain for the 60POE650/42 system at different temperatures, consistent with Refs. [28,31,55,56]. At low strain levels, the entanglement parameter shows a slight decrease at all four temperatures, indicating that chain relaxation and disentanglement occur gradually, primarily constrained by thermal motion and structural relaxation. However, as the strain increases beyond approximately 0.4, the entanglement parameter declines almost linearly, reflecting rapid inter- and intramolecular disentanglement.

**Figure 14:**
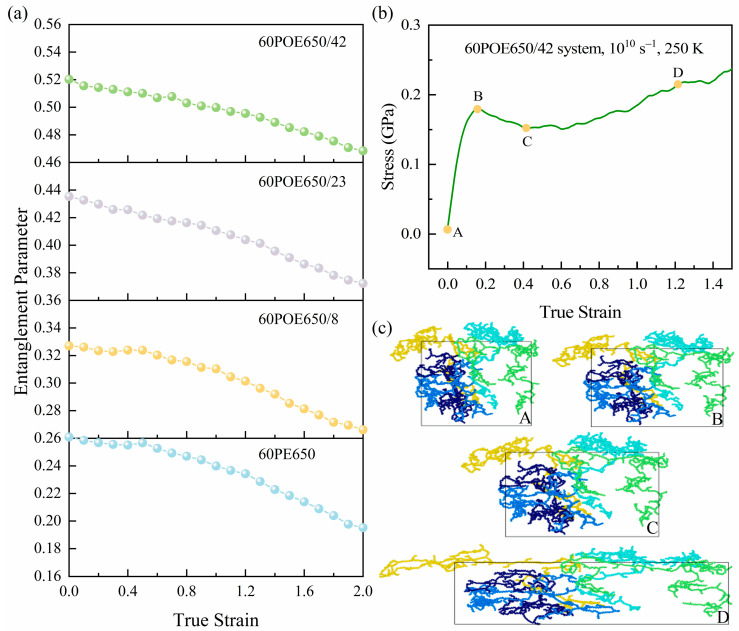
(**a**) The variation in chain entanglement parameters in response to strain for POE systems with varying hexyl branch contents at 250 K and 10^10^ s^−1^; (**b**) the stress–strain curve of the 60POE650/42 system under uniaxial tension at 250 K and 10^10^ s^−1^; and (**c**) the changes in polymer chain structures as shown by the stress–strain curve in (**b**).

To demonstrate how branch content affects the POE system’s material behavior during tensile deformation, the variation in chain entanglement parameters with strain in POE systems with different branch contents was calculated, as shown in Figure 14a. The variation in chain entanglement parameters during stretching differs for ethylene/1-octene copolymers with different branch contents. This is particularly evident as there is little to no significant decrease in the chain entanglement parameter at low strains for POE systems with low branch content or for linear polyethylene (60PE650). However, when the branch content of POE is higher, as exemplified by 60POE650/42 or 60POE650/23, the decrease in the chain entanglement parameter is more pronounced early in tensile deformation. Additionally, in Figure 14a, ethylene/1-octene copolymers have higher chain entanglement parameters compared to linear polyethylene, and the entanglement parameters increase with higher branch content. Figure 14c shows the evolution of the five molecular chain structures in the 60POE650/42 system at 250 K under tensile deformation, corresponding to the stress–strain graph shown in Figure 14b. All five molecular chains exhibit varying degrees of straightening as the strain increases, indicating the intramolecular disentanglement. However, even at high strain levels, intermolecular entanglements persist, suggesting that they are difficult to disentangle.

## 4. Conclusions

Molecular dynamics (MD) simulations were used to explore how amorphous ethylene/1-octene copolymers (POE) respond to uniaxial tensile loading. These simulations examined the effects of different strain rates, temperatures, chain lengths, chain numbers, and branch contents on the deformation response of the amorphous POE.

Strain rate, temperature, and branch content have a more pronounced impact on the deformation response compared to chain length and number. A notable difference in mechanical properties exists between linear polyethylene and ethylene/1-octene copolymers. Specifically, in the POE system, both the elastic modulus and the peak yield stress decrease as the hexyl branch content increases. During the elastic stage, van der Waals interactions are key contributors, with POE systems containing more branched chains experiencing larger changes in van der Waals energy. During strain softening and hardening, the transformation of dihedral angles is critical for the deformation of POE. As the branched chain content increases, the decrease in dihedral angle energy in the strain hardening regime becomes more gradual. Additionally, higher branched chain content results in a decreased rate and extent of the transition of dihedral angles to the *trans* conformation as deformation progresses. Ethylene/1-octene copolymers exhibit higher chain entanglement parameters compared to linear polyethylene, and these parameters increase with higher branch content. As strain increases, the chain orientation in the loading direction strengthens across all regimes. Moreover, the branched chain content has a significant impact on the change in chain orientation; in particular, higher branched chain content brings about a smaller increase in chain orientation. This study offers a comprehensive molecular-level understanding of how hexyl branch content influences the mechanical properties of ethylene/1-octene copolymers, which is essential for predicting polymer behavior across varying branch concentrations. By leveraging MD simulations to independently explore these effects, the study provides valuable insights that can inform experimental designs. Furthermore, the findings enable manufacturers to optimize material properties, enhancing performance in industries such as packaging, automotive, and biomedical applications, while improving elasticity, yield strength, and processing efficiency.

## Figures and Tables

**Table 1:** Bonded and non-bonded parameters applied in the MD simulations of ethylene/1-octene copolymers in this study.

Interaction Type	Parameters
Bond Length	*K*_bond_ = 350 kcal/mol, *r*_0_ = 1.53 Å
Bond Angle	*K*_angle_ = 60 kcal/mol/deg^2^, *θ*_0_ = 109.5°
Dihedral	*K*_0_ = 1.736, *K*_1_ = −4.490, *K*_2_ = 0.776, *K*_3_ = 6.990 (kcal/mol)
Non-Bonded	σ = 4.01 Å, ε = 0.112 kcal/mol

**Table 2:** POE systems with varying numbers of chains, chain lengths, and hexyl branch contents.

System	Chain Length, *l*_c_ ^a^	Molecular Weight *M*_w_, g·mol^−1^	Number of Chains, *n*_c_	Number of Branched Chains, *n*_BC_^b^	Content of Branched Chains, *c*_BC_/% ^c^
60PE650	650	9113	60	0	0
60POE650/8	650	10,094	60	9	8.31
60POE390/23	390	6730	60	15	23.07
60POE650/23	650	11,216	60	25	23.07
60POE1040/23	1040	17,946	60	40	23.07
30POE650/23	650	11,216	30	25	23.07
100POE650/23	650	11,216	100	25	23.07
60POE650/42	650	12,898	60	46	42.46

^a^ Chain length refers to the overall count of united atoms in one backbone structure. ^b^ *n*_BC_ is the number of hexyl branched chains in each POE chain. ^c^ *c*_BC_ is calculated as *c*_BC_ = 6 × *n*_BC_/*l*_c_ %, where *c*_BC_ represents the proportion of united atoms in all the branched chains of each POE chain relative to the total count of united atoms in the main chain.

**Table 3:** Elastic modulus and yield strength of POE systems with different branch contents under the current simulation and under real experimental conditions.

Current Simulation			Adapted from Ref. [16]		
Branch Content/wt%	E/MPa	σ_y_/MPa	Branch Content /wt%	E/MPa	σ_y_/MPa
0	1640	150	15.02	43.47	8.50
8.31	1390	141	20.50	18.31	6.50
23.07	1280	134	22.89	14.78	5.68
42.46	1160	120	24.57	13.73	5.23

**Table 4:** Elastic modulus and yield strength of the 60POE650/42 system at various temperatures and strain rates.

Temperature/K	Strain Rate έ/s^−1^	E/GPa	σ_y_/GPa
100	10^10^	2.40	0.23
250	10^10^	1.99	0.18
300	10^10^	1.16	0.12
300	10^9^	0.60	0.08
300	10^8^	0.26	0.04
400	10^10^	0.89	0.08

## Data Availability

The relevant data generated and analyzed in the current study are available from the corresponding author upon reasonable request.
